# Tibial condylar valgus osteotomy – indications and technique

**DOI:** 10.1186/s40634-020-00247-5

**Published:** 2020-05-13

**Authors:** Umito Kuwashima, Akihiko Yonekura, Masafumi Itoh, Junya Itou, Ken Okazaki

**Affiliations:** 1 Department of Orthopaedic Surgery, Tokyo Women’s Medical University, 8-1 Kawada-cho, Shinjuku-ku, Tokyo, 162-8666 Japan; 2 Department of Orthopaedic Surgery, Nagasaki University Graduate School of Biomedical Sciences, 7-1 Sakamoto, Nagasaki, 852-8501 Japan

**Keywords:** Tibial condylar valgus osteotomy, Medial unicompartmental osteoarthritis, Varus deformity, Joint laxity, L-shaped osteotomy

## Abstract

**Purpose:**

To describe the indications for, and surgical technique of, tibial condylar valgus osteotomy (TCVO).

**Indications:**

TCVO is commonly performed in patients with middle-to-end-stage medial unicompartmental osteoarthritis. Among the most important TCVO indication criteria are the types of tibial plateau shape. The convex-type (also called “pagoda-type”), with over a 5° joint line convergence angle on the standing X-ray, meets the indication criteria for TCVO.

**Surgical technique:**

An L-shaped osteotomy is performed from the medial side of the proximal tibia to the lateral beak of the intercondylar eminence. The apex of the L-shaped osteotomy line is on the medial border of the patellar tendon insertion. Surgeons should note the direction of the chisel (during the osteotomy) to the intercondylar eminence following fluoroscopic guidance. The posterior cortical bone is cut under a lateral view observation, and the crossed-leg position is adopted to prevent injury to the popliteal blood vessels. The spreader should be positioned at the posterior cortical bone to avoid increasing the tibial slope. The locking plate reliably stabilizes the osteotomy and helps shorten the period of postoperative rehabilitation.

**Conclusions:**

TCVO adjusts varus deformity alongside joint congruity. Accurate identification of indications and a detailed surgical plan would ensure effective correction and proper alignment. Additional osteotomies are recommended in case of under-correction of the varus limb deformity. TCVO is an effective intervention in patients with advanced knee osteoarthritis and lateral joint laxity with the pagoda-type tibial plateau shape.

## Background

Open-wedge high tibial osteotomy (OWHTO) is an established treatment for osteoarthritis (OA) with varus knee; patients are allowed early full weight-bearing using a locking plate, and several studies have reported desirable clinical outcomes after OWHTO [6, 20, 21]. Nevertheless, intra-articular deformities and large joint line convergence angles (JLCAs) occur in cases of severe varus knee OA. In such cases, the JLCA changes after valgus correction. Despite the proper surgical correction, change in JLCA can result in misalignment (overcorrection), and large preoperative JLCA is a risk factor for overcorrection [7, 15, 18]. Such large JLCAs can be observed in patients with advanced OA.

Tibial condylar valgus osteotomy (TCVO) is a type of OWHTO that was developed by Chiba (hence, “Chiba osteotomy”) [4]. The strategy of TCVO is to correct alignment and achieve joint stability and congruity. TCVO adjusts not only the varus deformity but also the joint congruity using an L-shaped osteotomy from the medial side of the proximal tibia to the lateral intercondylar eminence, and by correcting the knee alignment from varus to valgus. Recently, Chiba et al. reported good clinical outcomes of TCVO with short-term follow-up [5]. Nevertheless, only a few reports have described the indications and surgical technique employed in TCVO [9, 14, 23]. The appropriate indications and surgical techniques are important to achieve satisfactory results following a procedure of TCVO. Therefore, this report described the indications and surgical techniques for TCVO.

### Indications for TCVO

**Fig. 1:**
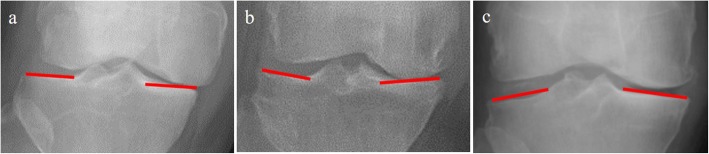
The types of tibial plateau shape: **a** Flat-type, **b** Depression-type, **c** Pagoda-type

TCVO is commonly performed in patients with middle-to-end-stage medial unicompartmental OA, classified as Kellgren–Lawrence grades III or IV [11]. Among the most important TCVO indication criteria are the types of tibial plateau shape. These are classified as follows [23]: flat-type, depression-type, and convex-type (also called “pagoda-type,” derived from the stupas in Myanmar) [17] (Fig. 1). The pagoda-type, including the tibial plateau shape of Blount disease, is indicated for TCVO because this knee type generally has larger laxity of the lateral compartment, and tends to be inaccurately corrected by OWHTO. The flat-type might be indicated for TCVO if there would be excessive joint laxity; flat-type knees with less laxity tend to be undercorrected by TCVO. Therefore, the indication of TCVO for the flat-type with varus OA remains controversial, and further study is needed to establish the optimal indication for those knee types. However, the depression-type is a contraindication for TCVO [23].

Large preoperative JLCA is another important factor involved in indications for the procedure. Typically, a JLCA over 5° on the standing X-ray with the pagoda-type, fulfills the indication criteria for TCVO. Regarding the range of motion (ROM), the indication of TCVO includes flexion contracture less than 10° and flexion over 90°. It is difficult to improve flexion contractures using TCVO. For this reason, excessive flexion contracture is a contraindication. In terms of age, there are no upper limits for TCVO. Generally, high tibial osteotomy (HTO) is performed in relatively younger active patients. However, recent studies indicated that age has no significant impact on the functional outcome after HTO [9, 13]. In addition, TCVO is performed in elderly patients approximately 65 years of age, with high activity. Notably, surgeons should consider the levels of patient activity and expectations.

### Surgical technique of TCVO

#### Preoperative planning

**Fig. 2:**
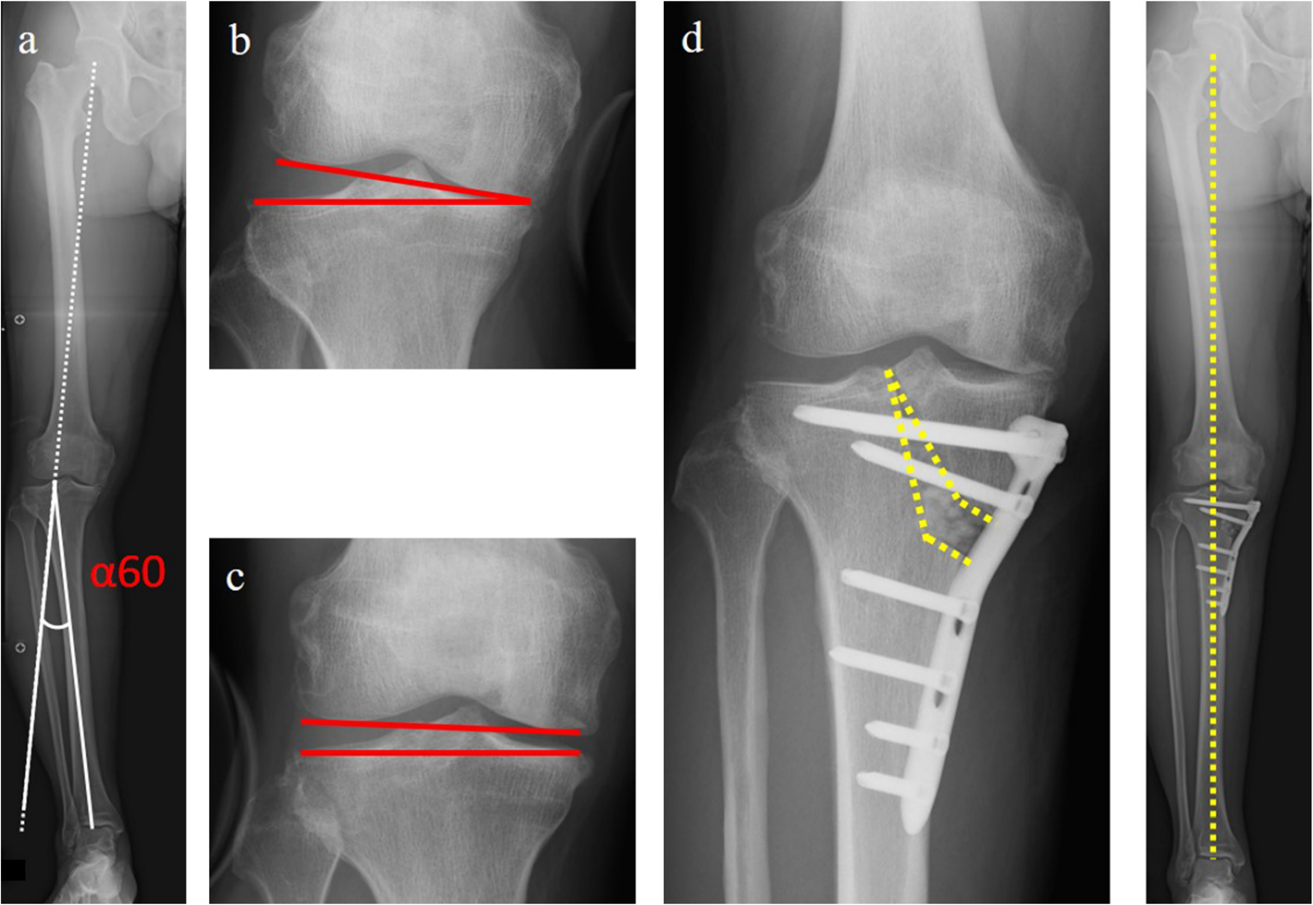
**a** Preoperative planning; α60 shows the correction angle calculated as the postoperative WBL passed through the 60% point of %MA. A hinge point is set to the lateral tip of intercondylar eminence. **b** Joint line convergence angle with varus stress X-ray. **c** Joint line convergence angle with valgus stress X-ray. β shows the value obtained by multiplying the sum of both JLCAs by 1.5. In this case, α60 was 11°, and β was 12° (varus- and valgus stress JLCA were 9° and − 1°, respectively). The value of β was greater than α60; thus, TCVO was performed to achieve the optimal alignment (**d**). JLCA: joint convergence angle. TCVO: tibial condylar valgus osteotomy

**Fig. 3:**
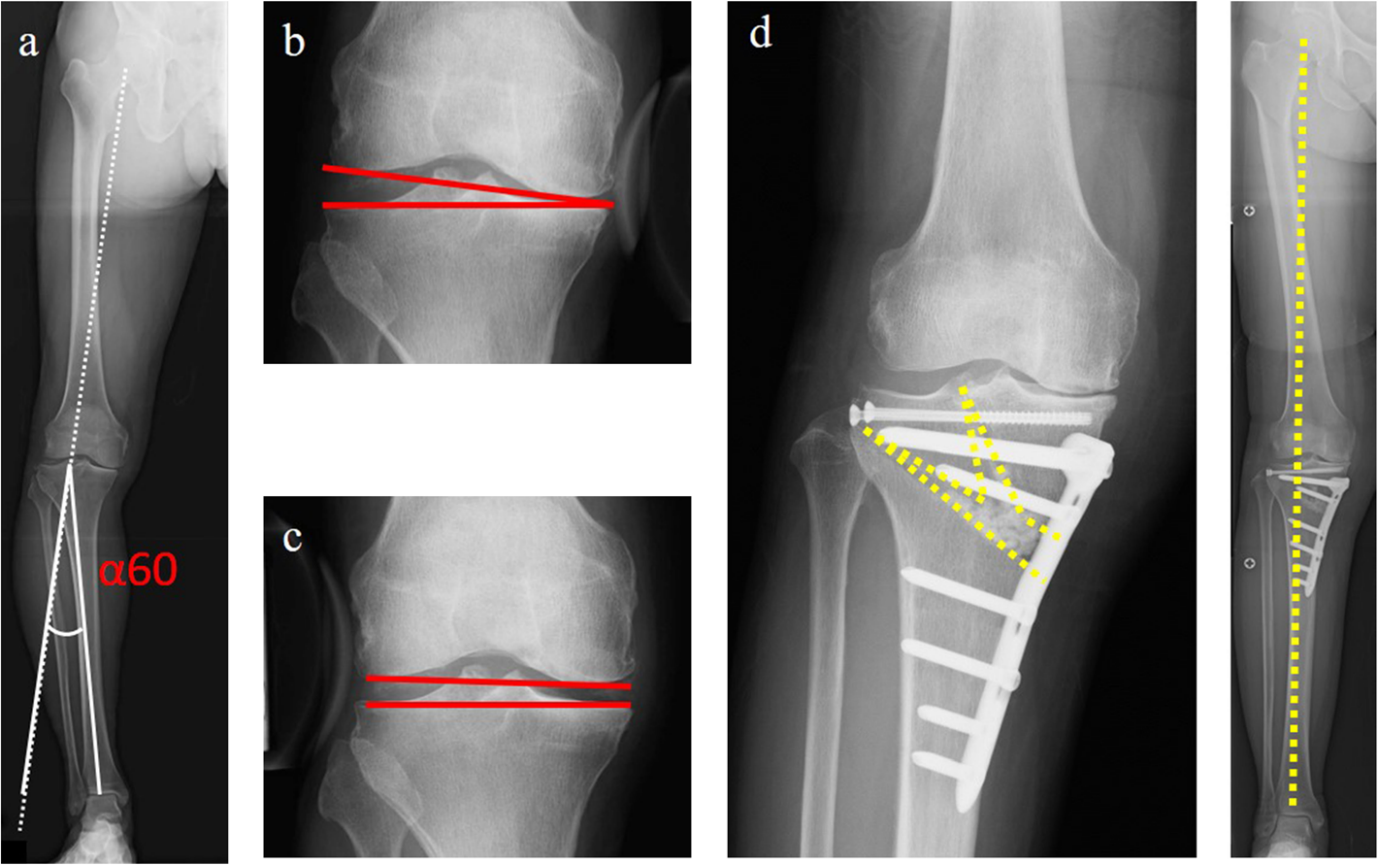
In this case, α60 was 14° (**a**) and β was 7.5° (**b**) varus stress JLCA was 6° and (**c**) valgus stress JLCA was − 1°). The value of β was smaller than α60; thus, TCVO and OWHTO were performed to achieve the optimal alignment (**d**). JLCA: joint convergence angle. TCVO: tibial condylar valgus osteotomy. OWHTO: open wedge high tibial osteotomy. α60: The correction angle calculated as the postoperative WBL passed through the point of 60%. β: The value obtained by multiplying the sum of both JLCAs by 1.5

Preoperative planning is crucial for TCVO and other osteotomies. The combined joint laxity with varus and valgus stress is close to the source of correction in TCVO. The percentage of the mechanical axis (%MA) is examined on standing full-length X-ray; %MA is defined as the ratio of the distance from the point of the weight-bearing line (WBL) at the knee joint to the medial edge of the tibial plateau and the width of the tibial plateau. The correction angle is calculated as the postoperative WBL passing through the 60% point of %MA [23]. A hinge point is set to the lateral tip of intercondylar eminence. In addition, the first line is drawn from the lateral tip of intercondylar eminence to the ankle center, and the second line is drawn from the lateral tip of intercondylar eminence to the line passing through the 60% point of %MA, which is the same length as the first line. The angle between the two lines is defined as the α60. Similarly, the JLCAs on varus and valgus stress X-rays are measured. The expected correction angle by TCVO (β) was calculated as follows: (varus stress JLCA + valgus stress JLCA) × 1.5 = β [23]. The term of “1.5” is empirically derived based on the soft tissue balance around the knee. The correlation between α60 and β is a crucial point in the determination of the surgical procedure. If β were greater than α60, TCVO would be sufficient to correct the target valgus alignment (Fig. 2). On the contrary, under-correction could occur when only TCVO is performed if β were less than α60. In these cases, additional OWHTO and/or distal femoral osteotomy (DFO) would be needed to achieve optimal alignment (Fig. 3).

#### Intraoperative technique

A skin incision is made from the point of the anterior-posterior center and 1 cm distal through the medial joint line to the distal tibial tuberosity. Half the proximal part of the pes anserinus was released. The tibial insertion of the medial collateral ligament is completely detached from the site of osteotomy to the distal end.

**Fig. 4:**
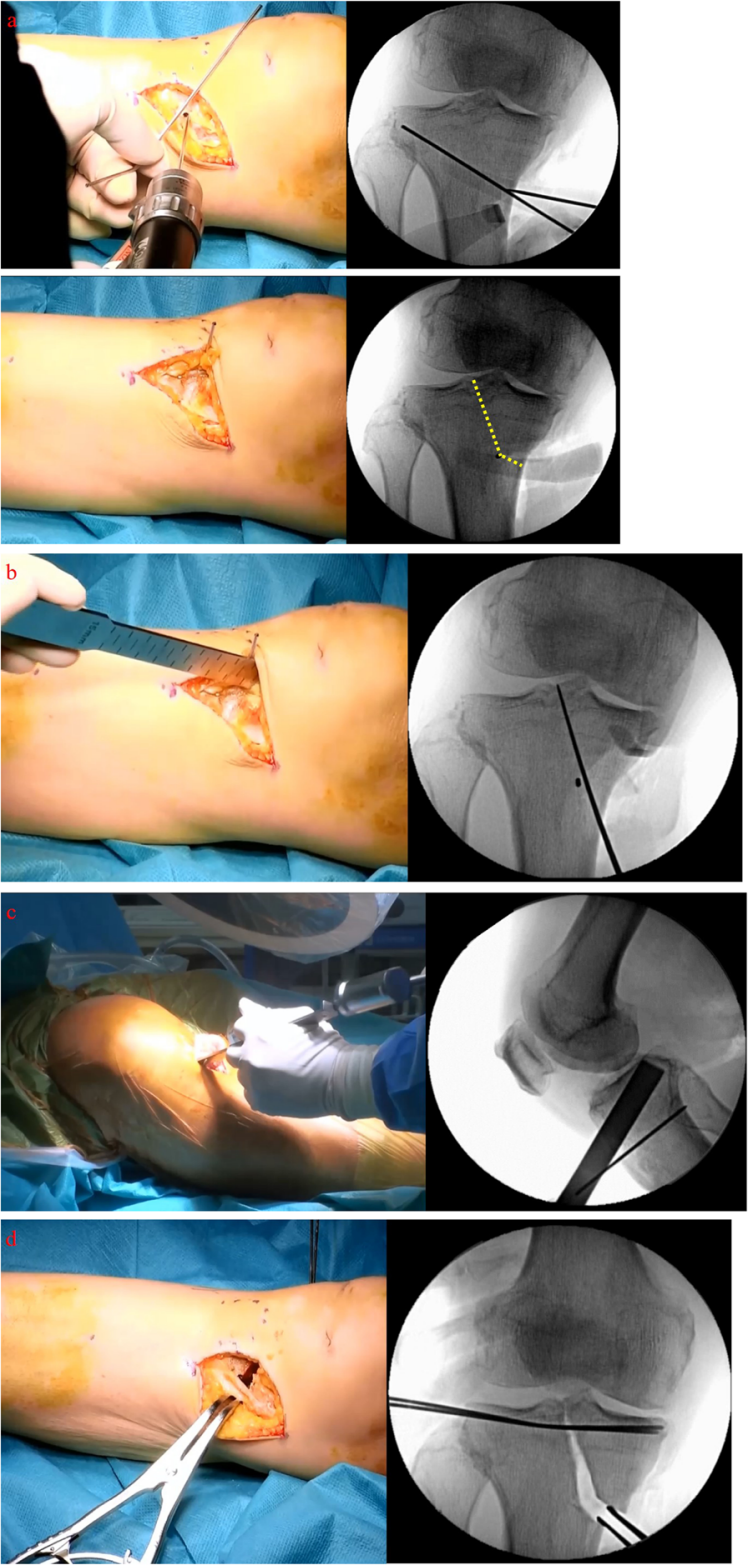
**a** This apex of L-shaped osteotomy is placed on the line connecting the transverse point and the apex of the fibula head, and a K-wire is inserted into the apex point of L-shaped osteotomy line; Dotted line: L-shaped osteotomy line. **b** The osteotomy to the intercondylar eminence is performed with a chisel following the fluoroscopic guidance. **c** The posterior cortical bone is cut following the fluoroscopic lateral view in a crossed-leg position. **d** Valgus correction is performed using the spreader. Two K-wires are inserted from the lateral side of the tibia plateau before the valgus correction

The location of the L-shaped osteotomy is marked. Initially, the transverse point is marked 4 cm distal from the medial joint line. The apex of the L-shaped osteotomy line is on the medial border of the patellar tendon insertion on the tibial tuberosity. This point is on the line connected to the transverse point and the apex of the fibula head for additional open wedge HTO, if needed. A 2.0-mm Kirschner wire (K-wire) is inserted into the apex point of the L-shaped osteotomy line from the anterior cortex to the posterior cortex (Fig. 4a). The osteotomy to the intercondylar eminence is performed using a chisel following fluoroscopic guidance. The chisel is inserted toward the lateral tip of the intercondylar eminence, cutting the anterior and superior cortical bones in the anteroposterior view. The chisel is inserted at an angle of 30° to the anterior tibial surface, and the insertion angle is increased up to 60° (Fig. 4b). Subsequently, the posterior cortical bone is cut following a fluoroscopic lateral view in a crossed-leg position to prevent injuring popliteal blood vessels (Fig. 4c). After the longitudinal cut, the transverse cut is performed as in OWHTO, using a bone saw and chisel.

Two 1.5-mm K-wires are inserted below and approximately parallel to the tibia plateau before the valgus correction, preventing hinge instability during the correction. Valgus correction is carefully performed using a spreader (Fig. 4d). The spreader should be positioned at the posterior cortical bone to avoid a tibia slope increase. The opening width of the osteotomy can be estimated by preoperative planning. A T-shaped locking plate is used for the fixation; however, the TomoFix™ plate (DePuySynthes, Solothurn, Switzerland) or a similar-shaped locking plate can be used. If needed, the plate is bent, depending on the shape of the proximal tibia. Proximal screws should be inserted beyond the center of the proximal tibia. The artificial bone substitute of β-TCP (OSferion: Olympus Terumo Biomaterials, Tokyo, Japan) can be used in the opening gap if a large correction is needed.

#### Postoperative rehabilitation

Active and passive ROM exercises are commenced on postoperative day 1. Full weight-bearing is generally allowed 1 week after surgery, depending on the severity of pain when only TCVO is performed. The patients who undergo additional osteotomies are assigned a longer period of partial weight-bearing, depending on the surgery. Patients who undergo TCVO and OWHTO are recommended partial weight-bearing for 2 weeks after surgery.

## Discussion

The primary advantages of TCVO are as follows: (1) joint congruity is enhanced, resulting in improvement of joint stability; (2) early weight-bearing can be commenced with the locking plate because the lateral tibial condyle is intact; and (3) the adverse effects on the patellofemoral joint may be minimal compared to those associated with OWHTO.

Clinical outcomes of HTO are not always satisfactory in patients suffering from severe degenerative or unstable OA knee with lateral thrust [2, 3]. Coronal tibiofemoral subluxation of the tibia is related to postoperative under-correction [1, 19]. TCVO alters the shape of the tibial joint surface alongside the limb alignment; however, it can adjust the congruity of the medial and lateral knee joints and can provide load redistribution. Remarkably, JLCA can be corrected after TCVO. A previous study reported a postoperative marked decrease in the JLCA of TCVO group relative to the OWHTO group [5], which provided joint stability. Furthermore, because the shape of the tibial plateau becomes more concave, the femoral condyle is wedged from both sides; therefore, joint stability can be simultaneously achieved [14].

Recently, several studies reported progressive degeneration in the patellofemoral joint (PFJ) after OWHTO [8, 12, 22]. The change in patellofemoral alignment or the progress of OA after OWHTO did not significantly affect the clinical outcomes in mid-term follow-up [8, 16]. Nevertheless, long-term effects have been unclear. Because the change in the height of tibial tuberosity with TCVO is less than that associated with OWHTO, the effect on the PFJ after TCVO is supposedly minimal compared to that of OWHTO [14].

A few previous studies have assessed the radiographic and clinical outcomes of TCVO. Higuchi et al. evaluated the radiological features of TCVO [10]. It was observed that TCVO provided the preferable postoperative limb alignment, and reduced the mean varus stress angle from 7.2° to 4.0°; TCVO equally reduced the laxity angle (defined as the total amplitude of varus- and valgus-stress angle) from 9.5° to 4.5°, without any ligament reconstructions. Chiba et al. reported clinical outcomes of TCVO with a 5-year follow-up [5]. Ten cases involving 11 knees with medial OA and a spread lateral joint were treated using TCVO. The demographic data of participants were as follows (mean ± deviation): mean age, 57 ± 6 years; 3 males and 7 females; body mass index, 29.0 ± 2.7 kg/m^2^; and Kellgren–Lawrence grade III (6 participants) and IV (5 participants). In clinical evaluation, a visual analog scale (VAS), Western Ontario and McMaster Universities (WOMAC) score, and ROM of the knee were evaluated. Postoperatively, VAS improved from an average of 73 mm to 13 mm, and the total WOMAC score similarly improved from 52 to 14 at 5 years postoperatively. The ROM at the final follow-up was equivalent to preoperative values. The mechanical axis changed from 1% to 60%, and JLCA changed from 6° to 1° after TCVO. Pain and activities of daily living, as well as the joint instability in the coronal plane, were improved after TCVO even in advanced OA knees.

The disadvantage of TCVO is the limited angle of valgus correction. The change in coronal alignment after TCVO is associated with the changes in the JLCA. If a further valgus correction is needed, OWHTO and/or DFO should be considered alongside TCVO to achieve optimal alignment. Preoperative planning is important in predicting the need for such additional osteotomies.

In conclusion, TCVO adjusts not only the varus deformity but also joint congruity. Indications are crucial to achieving accurate alignment. In the event varus limb deformity is undercorrected, additional osteotomies (OWHTO/DFO) should be similarly performed. Overall, TCVO is an effective intervention for patients with advanced knee osteoarthritis and lateral joint laxity with the pagoda-type of a tibial plateau shape. However, further study is needed to establish the optimal indication for other types of knees and the appropriate JLCA correction.

## Data Availability

Data sharing is not applicable to this article, as no datasets were generated or analyzed during the current study.

## Abbreviations

TCVO: Tibial condylar valgus osteotomy
OWHTO: Open-wedge high tibial osteotomy
OA: Osteoarthritis
JLCA: Joint line convergence angle
%MA: percentage of mechanical axis
WBL: Weight-bearing line
DFO: Distal femoral osteotomy
K-wire: Kirschner wire
HTO: High tibial osteotomy
ROM: Range of motion
PFJ: Patellofemoral joint
